# Vitamin D and uric acid: Is parathyroid hormone the missing link?

**DOI:** 10.1016/j.jcte.2021.100263

**Published:** 2021-07-08

**Authors:** Ben Ponvilawan, Nipith Charoenngam

**Affiliations:** a Department of Pharmacology, Faculty of Medicine Siriraj Hospital, Mahidol University, Bangkok, Thailand; b Department of Medicine, Faculty of Medicine Siriraj Hospital, Mahidol University, Bangkok, Thailand; c Section Endocrinology, Diabetes, Nutrition and Weight Management, Department of Medicine, Boston University School of Medicine, Boston, MA, United States; d Department of Medicine, Mount Auburn Hospital, Harvard Medical School, Cambridge, MA, United States

**Keywords:** Vitamin D, Uric acid, Hyperuricemia, Parathyroid hormone, PTH

Dear Editor,

With interest, we read the article of Nimitphong et al. [1], which revealed the potential uric-lowering effect of vitamin D supplementation in patients with prediabetes and hyperuricemia. The result of this study supports the causal association between vitamin D deficiency and insufficiency and elevated serum uric acid that has been shown in previous observational studies [2] independent of the fact that the association might also be confounded by presence of comorbid obesity and metabolic syndrome.

We would like to highlight that the uric acid-lowering effect of vitamin D supplementation is likely mediated by the decrease in serum parathyroid hormone (PTH) level. Circulating 25(OH)D gets metabolized by the enzyme 1α-hydroxylase in the kidney into 1,25-dihydroxyvitamin D [1,25(OH)_2_D], which inhibits PTH synthesis directly by the activation of vitamin D receptor in the parathyroid glands and indirectly by stimulating intestinal calcium absorption causing a transient increase in serum ionized calcium level [3]. The authors did mention that vitamin D might affect the expression of uric acid-regulating genes. In fact, it is known that PTH could directly downregulate the ATP-binding cassette transporter G2 (ABCG2) transporter, causing a reduction in urinary uric acid excretion [4]. Moreover, it has been shown that patients with primary hyperparathyroidism had increased levels of serum uric acid and that those patients who received parathyroidectomy also had decreased serum uric acid levels postoperatively [5], [6], [7]. All of these, along with the evidence that teriparatide [PTH(1–34)] could induce hyperuricemia, would therefore support the causal association of hyperparathyroidism with decreased renal clearance of uric acid and hyperuricemia. Therefore, it would be of great interest to further investigate if the decrease in uric acid level after vitamin D supplementation is secondary to increased urinary uric acid excretion and if this change is dependent on the decrease in serum PTH level.

As mentioned by the authors, uric acid is shown to suppress the expression of the *CYP27B1* gene which encodes the enzyme 1α-hydroxylase in the kidney *in vitro* and *in vivo*, leading to decreased conversion of 25(OH)D into 1,25(OH)_2_D [8]. It is therefore worth noting that vitamin D, PTH and uric acid forms a cycle of positive feedback, as shown in the Fig. 1.

**Fig. 1:**
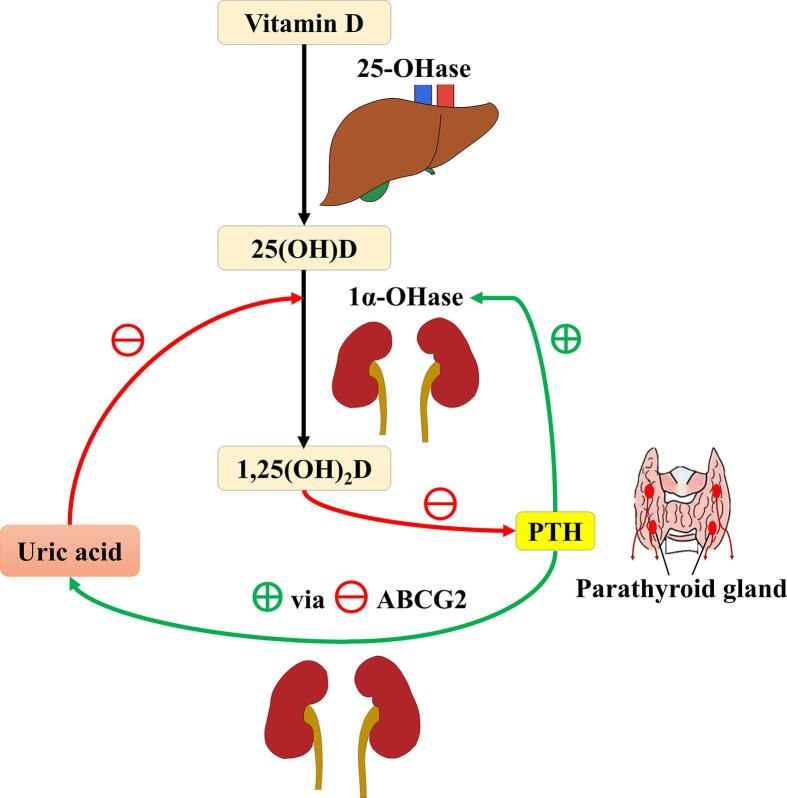
Schematic representation of the interplay of vitamin D, parathyroid hormone and uric acid. Uric acid inhibits the expression of the *CYP27B1* gene, which encodes the enzyme 1α-hydroxylase that converts 25-hydroxyvitamin D into 1,25-dihydroxyvitamin D at the renal proximal tubule. 1,25-dihydroxyvitamin D inhibits parathyroid hormone secretion at the parathyroid glands directly and indirectly by stimulating intestinal calcium absorption leading to a transient increase in serum ionized calcium. Parathyroid hormone inhibits the ATP-binding cassette transporter G2 transporter, which, in turn, results in decreased urinary uric acid excretion and hyperuricemia. Abbreviations: 1α-OHase: 1α-hydroxylase; 25-OHase: 25-hydroxylase; 1,25(OH)_2_D: 1,25-dihydroxyvitamin D; 25(OH)D: 25-hydroxyvitamin D; ABCG2: ATP-binding cassette transporter G2 transporter; PTH: parathyroid hormone.

Finally, we propose that elevation of serum uric acid may be a marker of vitamin D deficiency and/or hyperparathyroidism and that screening and treating vitamin D deficiency may be warranted in patients with hyperuricemia. Further study should be conducted to determine the clinical utility of this measure.

## Funding

Nipith Charoenngam receives the institutional research training grant from the Ruth L. Kirchstein National Research Service Award program from the 10.13039/100000002National Institutes of Health (2 T32 DK 7201-42).

## Authors’ contributions

All authors had a role in writing the letter.

## Declaration of Competing Interest

The authors declare that they have no known competing financial interests or personal relationships that could have appeared to influence the work reported in this paper.
